# Stable Display of Artificially Long Foreign Antigens on Chimeric *Bamboo mosaic virus* Particles

**DOI:** 10.3390/v13040572

**Published:** 2021-03-29

**Authors:** Tsung-Hsien Chen, Chung-Chi Hu, Chin-Wei Lee, Yu-Min Feng, Na-Sheng Lin, Yau-Heiu Hsu

**Affiliations:** 1 Graduate Institute of Biotechnology, National Chung Hsing University, Taichung 40227, Taiwan; cych13794@gmail.com (T.-H.C.); cchu@dragon.nchu.edu.tw (C.-C.H.); wei570211@yahoo.com.tw (C.-W.L.); 2 Department of Internal Medicine, Ditmanson Medical Foundation Chia-Yi Christian Hospital, Chiayi 60002, Taiwan; fengyumin2@gmail.com; 3 Advanced Plant Biotechnology Center, National Chung Hsing University, Taichung 40227, Taiwan; 4 Division of Gastroenterology and Hepatology, Department of Internal Medicine, Ditmanson Medical Foundation Chia-Yi Christian Hospital, Chiayi 60002, Taiwan; 5 Institute of Plant and Microbial Biology, Academia Sinica, Taipei 11529, Taiwan; nslin@sinica.edu.tw

**Keywords:** *Bamboo mosaic virus*, longer antigens, chimeric virus particles (CVPs), flexible linker, peptide properties, virus accumulation

## Abstract

Plant viruses can be genetically modified to generate chimeric virus particles (CVPs) carrying heterologous peptides fused on the surface of coat protein (CP) subunits as vaccine candidates. However, some factors may be especially significant in determining the properties of chimeras. In this study, peptides from various sources and of various lengths were inserted into the Bamboo mosaic virus-based (BaMV) vector CP N-terminus to examine the chimeras infecting and accumulating in plants. Interestingly, it was found that the two different strains Foot-and-mouth disease virus (FMDV) VP1 antigens with flexible linker peptides (77 or 82 amino acids) were directly expressed on the BaMV CP, and the chimeric particles self-assembled and continued to express FMDV antigens. The chimeric CP, when directly fused with a large foreign protein (117 amino acids), can self-fold into incomplete virus particles or disks. The physicochemical properties of heterologus peptides N-terminus, complex strand structures of heterologus peptides C-terminus and different flexible linker peptides, can affect the chimera accumulation. Based on these findings, using plant virus-based chimeras to express foreign proteins can increase their length limitations, and engineered plant-made CVP-based vaccines have increasing potential for further development as novel vaccines.

## 1. Introduction

Molecular agricultural technology, utilizing plants as bioreactors to produce valuable proteins, has increased in recent years [1,2,3]. Among the commonly used approaches, virus-based transient expression vector systems are the most promising for rapid recombinant protein expression, having higher potential than stable transgenic plants [2]. Among plant virus-based vectors, through the epitope presentation system, plant-made chimeric virus particles (CVPs) displaying pathogenic antigens on their surfaces as fusions to the coat proteins (CPs) can be potential vaccine candidates [2,3,4,5]. The CVP strategy has been used extensively to display target peptides on the CVP surface to enhance immunogenicity [6,7]. CPs can enhance immunogenicity [6,8] and facilitate easy antigen purification [5]. Additionally, when intranasally, intraperitoneal or oral plant-made CVP immunization is administered to animals, they can induce strong neutralizing immune responses and protect against diseases [1,5,9,10,11,12]. Hence, plant-made CVPs have the potential to be developed into vaccines against various diseases.

However, CVP production in plants has several limitations [13]. Directly displaying the foreign peptides on the rod-shaped CVP surface, differing peptide lengths [12,14], isoelectric point (pI) [15,16,17], charge [17] and the content of tryptophan [16,18], threonine [16,18], serine [16,18], or cysteine [19], can affect CVP infectivity, morphology, stability, virus-host interactions and long-distance movement. Cowpea mosaic virus (CPMV)-based vectors directly express foreign peptides on icosahedral CVP surfaces, and their peptide length and pI affect CPMV CVP symptoms, phenotypes and yields [20]. The special structural features of the chimeric CP may prevent self-assembly into CVPs [21] and has the tendency to delete insertions, eventually reverting back to a wild-type virus after several cycles of infection [16,20]. Therefore, further studies to investigate the effects of foreign peptide characteristics on the ability of recombinant viruses to generate CVPs are important.

Bamboo mosaic virus (BaMV) is a filamentous rod consisting of single-stranded positive-sense RNA. BaMV CP reveals flexible N- and C-terminal extensions, allowing deformation while maintaining structural integrity [22]. The 35 amino acids in the BaMV CP N-terminus contain a unique glycine-rich motif-containing region, the deletion of which has no effect on the virus characteristics [22,23]. Therefore, the BaMV CP N-terminus, which removes the 35-amino acid vector, BS-d35CP, has been successfully developed as an epitope presentation system, and directly expressed peptides of up to 37 amino acids [9]. Previous reports have demonstrated that a BaMV-based vector is an effective epitope presentation system [9,11]. The foot-and-mouth disease virus (FMDV) VP1 epitopes expressed on BaMV CVP surfaces effectively induce humoral and cell-mediated immune responses in swine and provide full protection against FMDV challenges [9]. Following intramuscular immunization with very virulent infectious bursal disease virus (vvIBDV) VP2 epitopes expressed on BaMV CVP surfaces, chickens produced antibodies against IBDV and were protected from vvIBDV (V263/TW strain) challenges [11]. These results indicate that it is possible to successfully produce vaccine candidates based on the BaMV CVP platform using a plant cell suspension culture system instead of conventional bacterial or animal cell culture systems [24]. Therefore, this report is the first to determine whether the physical and chemical parameters and structure of heterologous peptides affect the accumulation of BaMV CVP. Second, we aimed to explore the effect of large heterologous peptides with flexible linkers directly fused onto BaMV CPs on virion assembly and stability.

## 2. Materials and Methods

### 2.1. Construction of Chimeric Virus Infectious Clones

**Figure 1:**
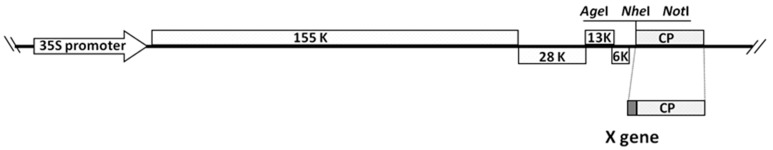
Schematic representation of the recombinant constructs based on the bamboo mosaic virus-based (BaMV) genome. pBS-d35CP is a mutant infectious construct of BaMV with N-terminus of the 35 amino acids truncated coat protein (CP) under the control of cauliflower mosaic virus 35S promoter. The restriction enzyme recognition sites for cloning are indicated on top.

The infectious recombinant constructs were constructed by inserting various polypeptide nucleotide sequences at the 5′-terminus of the truncated CP open reading frame of the vector pBS-d35CP [9] following digestion with the appropriate restriction enzyme (Figure 1). Detailed methods are provided in the supplementary methods (Methods S1). All plasmids were confirmed by nucleotide sequencing.

### 2.2. Physicochemical Parameters Prediction of *Foreign Peptides*

The physical and chemical properties of the peptides were calculated using the ProtParam program on the ExPASy server (http://web.expasy.org/protparam/; accessed on 8 January 2012). These computed parameters include the theoretical pI, number of charged residues and grand average of hydropathicity (GRAVY; positive GRAVY index, hydrophobic; negative GRAVY index, hydrophilic). The computational predictions of disulfide bond connectivity were estimated using the DiANNA 1.1 web server [25] to predict hypothetical chimeric CP disulfide bonds. Protein secondary structures were simulated using the PSIPRED protein structure prediction server [26].

### 2.3. Plant Inoculation and Detection of Chimeric Virus Accumulation

Infectious cDNA clones of recombinant chimeric viruses were inoculated onto the local lesion host *Chenopodium quinoa* or the systemic infection host *Nicotiana benthamiana,* as previously reported [27]. These plants were grown in a greenhouse exposed to normal daylight. For the infectivity assay, infectious chimeric cDNAs were inoculated onto three *C. quinoa* or *N. benthamiana* (1 µg 10 μL^–1^ per leaf) plants with four or two fully expanded leaves by gently rubbing carborundum-dusted leaves with cotton swabs. The inoculated *C. quinoa* leaves were harvested at 10 days post infection (dpi). The inoculated *N. benthamiana* leaves and systemic-infected leaves were harvested at 14 and 21 dpi, respectively. Proteins extracted from the inoculated and systemic-infected leaves were separated using 12% SDS-PAGE, transferred to polyvinylidene fluoride (PVDF) membranes (Millipore, MA, USA) and reacted with antsera against BaMV CP [28], *influenza A virus* M2 (Abcam ab56086), FMDV VP1 [29], anti-FMDV VP1-97 (O/TAW/97, Methods S2), anti-FMDV VP1-99 (O/TAW/99, Methods S2), or JEV EDIII [12], respectively, to confirm the molecular identity of the chimeric viruses.

The accumulation levels of chimeric viruses were analyzed using an indirect enzyme-linked immunosorbent assay (ELISA). ELISA was performed as previously described, with minor modifications [9]. Total inoculated leaf protein extract dilutions were incubated overnight in ELISA plates (Nunc). Rabbit antisera against BaMV CP were then added to each well [28] and the bound protein antibodies were detected with biotin-conjugated goat antirabbit IgG using the VECTASTAIN Elite ABC kit (avidin biotinylated peroxidase; Vector Laboratories). Following color development, the absorbance at 450 nm was measured using an ELISA reader (Spectramax M2, Molecular Device, San Jose, CA, USA). A standard curve was created using solutions containing purified BaMV virion of a known concentration, and then compared with the generated ELISA data. Noninoculated leaf protein extract was used as a negative control.

### 2.4. Serologically Specific Electron Microscopy (SSEM) of CVPs

Methods used for the examination of chimeric BVP1s (various FMDV VP1 epitopes expressed on BaMV) virions by SSEM were performed as previously reported [30]. Copper grids were precoated with antisera specific for BaMV CP [28] at 1:100 dilution before the crude sap was dropped. Crude sap from freshly inoculated *C. quinoa* leaves at 10 dpi was collected and dropped onto glow-discharged carbon-coated copper grids. The grids were then negatively stained with 2% uranyl acetate and examined using transmission electron microscopy (Philips CM 100 Bio) at 80 kV. BaMV and BS-d35CP virions were examined using the same method as the control.

### 2.5. Infected Plant Tissue Protein Analysis and BVP1s Chimera Stability during Sequential Transmission

The stability of the BVP1s chimeric viruses was tested using *C. quinoa* and *N. benthamiana*. The infectious cDNA clones of pBS-d35CP, pBVP1 [9], pBVP1-9, pBVP1-7A7, pBVP1-7A9, and pBVP1-7AG9 were inoculated into *C. quinoa* and *N. benthamiana*, as previously reported [27]. After symptoms appeared on the pBVP1s-inoculated *C. quinoa* or *N. benthamiana* leaves at 10 dpi, the leaves were excised and mechanically transferred to healthy *C. quinoa* and *N. benthamiana*, respectively. The procedure mentioned above was repeated three times, and the progeny viruses BVP1-7A7, BVP1-7A9 and BVP1-7AG9 on *C. quinoa* and *N. benthamiana* leaves were assayed each time to examine the stability of the chimeric virus after successive passages in plants. Total protein contents were analyzed via Western blotting to confirm the stability of BVP1s.

### 2.6. Statistical Analysis

Differences between groups were evaluated using Student’s *t*-test, where *p* < 0.01, was considered statistically significant.

## 3. Results

### 3.1. Expression Efficiency of Chimeras from Different Recombinants Variations

To determine the ability of individual peptides to influence infectivity and chimeric virus accumulation, peptides of different lengths and amino acid compositions were selected to directly express foreign peptides on BaMV CVP surfaces. Heterologous peptide nucleotide sequences were constructed into the 5′-terminus of the CP plasmid pBS-d35CP (Figure 1), as described in previous studies [9,11]. A panel of polypeptides was selected from influenza A virus M2 extracellular domain (M2e), influenza A virus hemagglutinin (HA), influenza A virus HA2, FMDV VP1, T-cell epitopes, or Japanese encephalitis virus envelope protein domain III (JEV EDIII) epitopes (Table S1).

We determined the infectivity levels of the recombinant viruses in the local lesion host *C. quinoa*. Chimeric BaMV infectious clones developed symptoms at 10 dpi. To further verify that CP generated from chimeric BaMV-inoculated plants contained various antigenic epitopes, Western blotting analysis was performed using specific antisera against BaMV CP, influenza A virus M2, FMDV VP1, or JEV EDIII, respectively. The inoculated leaves were then examined to evaluate the presence of these CVP accumulations through ELISA. The chimeras were divided into six groups: no-infection, 1–20%, 21–40%, 41–60%, 61–80%, and 81–100% infection with BS-d35CP, respectively (Table S1 and Table 1).

### 3.2. Effects of Physicochemical Parameters of Heterologous Peptides

**Figure 2:**
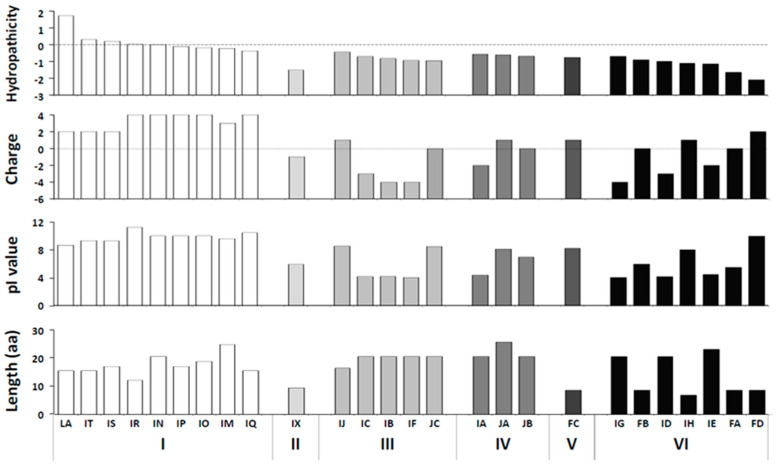
The effect amino acid physicochemical parameters of the heterologous peptide on chimeric virus accumulation. The bars show the hydropathicity, charged, pI value and length grand averages for foreign peptide encoded by each construct. Chimeric BaMV accumulation in *C. quinoa* was normalized using BS-d35CP (100%) to account for chimeras; group I: 0%, II: 1–20%, III: 21–40%, IV: 41–60%, V: 61–80%, VI: 81–100%. Group I, II, III, IV, V, and VI are distinguished using white to black shadings.

Hydrophobic amino acids play a key role in the self-assembly of protein molecules [31]. Potexvirus virion surfaces are highly hydrated, and the water molecules bound to these surfaces help to maintain the surface structure of the virions [32]. We evaluated the sum of the hydropathy values for all the amino acids based on the GRAVY score program, which are extensions of the BaMV CP, and the heterologous peptide hydrophilicity values, both of which are key factors for chimera infection and accumulation in plants (Table S1 and Figure 2).

The pI value of a protein molecule can be predicted by adding the number of positively charged residues and protonated amino termini and subtracting the number of negatively charged residues and deprotonated carboxyl termini. We estimated the pI value and charge of the heterologous peptides using the ProtParam program (http://web.expasy.org/protparam/; accessed on 8 January 2012) [33]. The results are shown in Table S1 and Figure 2. Foreign peptides containing excessive positive charges or high pI values affect the infective ability of chimeric viruses, and the lengths of heterologous peptides fused to the plant virus CPs are constraints on chimera accumulation, assembly and movement [20]. Therefore, the accumulated chimeras are not directly affected by chimeric viruses containing different heterologous peptide fragment lengths (within a certain range, Figure 2) but are dependent on other physicochemical factors. Therefore, the hydrophobicity, charge, pI value, fragment length and other physicochemical parameters of the heterologous peptides fused on chimera CPs were greatly different than those of the original CP, which could affect chimera infection and accumulation.

### 3.3. Secondary Structure Prediction

**Figure 3:**
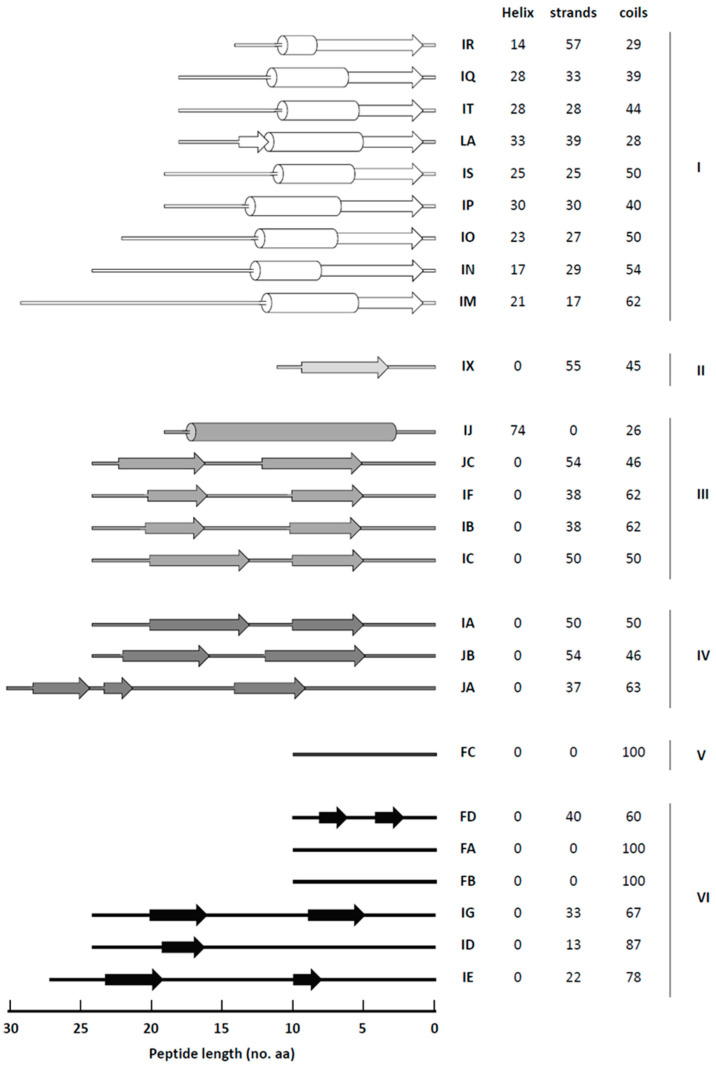
Foreign peptide secondary structures comparison. The chimeric BaMV CP N-terminus secondary structures contents are fused with various foreign peptide regions. Foreign peptide secondary structure assignments are determined by computer prediction with the PSIPRED server algorithm. β-strands are represented by arrows, α-helices are indicated represented by bars, and coil are represented by lines. Numbers represent the mean α-helices, β-strand, and coil in percentage in the foreign peptides, respectively. Chimeric BaMV accumulation in *C. quinoa* was normalized using BS-d35CP (100%) to account for chimeras; group I: 0%, II: 1–20%, III: 21–40%, IV: 41–60%, V: 61–80%, VI: 81–100%. Group I, II, III, IV, V, and VI are distinguished using white to black shadings. The length of foreign peptides is shown on the bottom.

Protein structure is determined by the amino acid sequence, as well as a multitude of interactions, localized within a secondary structural element, and points of contact in longer regions. Heterologous peptides fused to chimeric virus CPs that contain a high proportion of helix and strand structures, or peptide C-terminus-containing complex strand structures, have the potential to affect the infection and accumulation ability of the chimeras (Figure 3). Therefore, reducing the structural barriers of heterologous peptides may improve the length limitation of chimeric fusion fragments.

In addition, disulfide bonds form between the sulfur atoms of two cysteine side chains in a protein and may stabilize the folded state enthalpically through favorable local interactions. Intramolecular disulfide bonding is important for CP conformation, for example, potato virus X (PVX) [34], *sindbis virus* [35], and *betanodavirus* [36]. In this study, the wild-type BaMV-S CP was found to contain three cysteine residues (Cys-129, Cys-168 and Cys-181), and a disulfide bond was predicted to form between Cys-168 and Cys-181, according to the DiANNA 1.1 disulfide bond prediction program [25]. Based on the disulfide bond-predicting program results, we speculated that the cause of the difference in accumulation levels is that peptides IA, IB, IC, or IF change the original chimera CP disulfide bonds. These results show that the original chimera CP disulfide bond modification may inhibit chimeric virus accumulation.

### 3.4. The N-Terminus Effect of Heterologus Peptides on Chimeras

We identified the important features of foreign proteins fused on BaMV CP, such as hydrophilicity, pI values, charges, fragment lengths and secondary structure characteristics, which have obvious effects on the accumulation of chimeras. Two analogous polypeptide fragments from FMDV O serotype porciniphilic strain (O/TAW/97) VP1 epitopes, with a charge:pI:hydropathicity value of +1:8.10:-0.811, and from PanAsia strain (O/TAW/99) VP1 epitopes, with a charge:pI:hydropathicity value of +2:9.10:-0.468, were used. Furthermore, we added a flexible (AAA or AAAGGGGS) spacer [37] between the analogous FMDV O/TAW/97 VP1 epitopes and O/TAW/99 VP1 epitopes.

First, we compared the differences between pBVP1-7A9 and pBVP1-9A7, or pBVP1-7AG9 and pBVP1-9AG7 to determine the impact of N or C-terminus polypeptides on chimeric virus infection and accumulation. Second, the differences between pBVP1-7A9 and pBVP1-7AG9, or pBVP1-9A7 and pBVP1-9AG7 were compared to determine the impact of the flexible (AAA or AAAGGGGS) spacer or peptide length. BVP1-7A7 and BVP1-9A9 viruses were constructed as controls, and BVP1-9A7A7 was constructed from three fragments (Table 1).

### 3.5. Infection and Systemic Movement of the Chimeras with Heterologus Peptides

**Figure 4:**
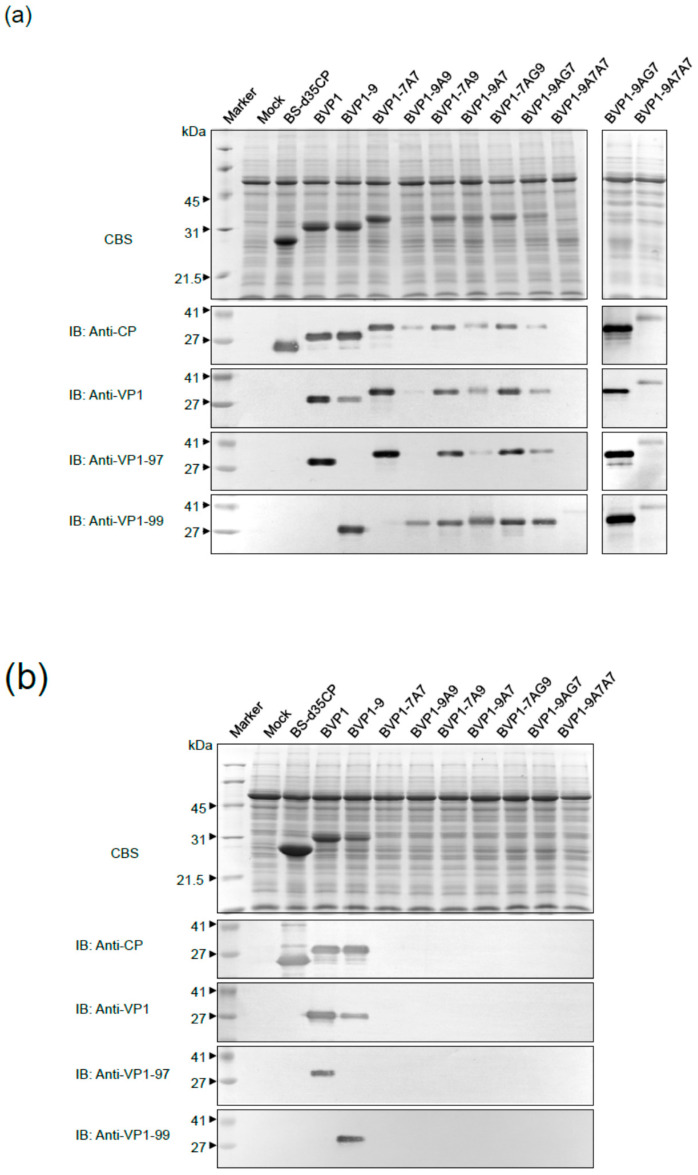
FMDV VP1 epitopes are expressed in *N. benthamiana* infected with chimeric BaMV. SDS-PAGE separation and immunoblot of proteins extracted from (**a**) inoculated or (**b**) systemically infected *N. benthamiana* leaves. Leaves were inoculated with H_2_O (mock) or with recombinant plasmid pBS-d35CP, pBVP1, pBVP1-9, pBVP1-7A7, pBVP1-9A9, pBVP1-7A9, pBVP1-9A7, pBVP1-7AG9, pBVP1-9AG7, and pBVP1-9A7A7, as indicated. Total proteins extracted from the inoculated leaves were separated using 12% SDS-PAGE gel (Top panel) and stained with Coomassie Blue stain (CBS). The proteins were transferred to polyvinylidene fluoride membranes and reacted with antisera against BaMV CP (anti-CP), FMDV VP1 (anti-VP1), FMDV VP1 (O/TAW/97 specificity, anti-VP1-97), or FMDV VP1 (O/TAW/99 specificity, anti-VP1-99), respectively. The relative molecular weights (in kDa) are given on the left of each panel. IB, immunoblot.

We further investigated the influence of the heterologous N or C-terminus peptide on the factors affecting chimera accumulation. To determine whether fusion proteins were produced during host systemic infection, *N. benthamiana* was inoculated with chimeric viruses. Total protein was extracted from the leaves inoculated with H_2_O (mock), pBS-d35CP, pBVP1, pBVP1-9, pBVP1-7A7, pBVP1-9A9, pBVP1-7A9, pBVP1-9A7, pBVP1-7AG9, pBVP1-9AG7, or pBVP1-9A7A7, and were subjected to SDS-PAGE (Figure 4a) and Western blotting assays using BaMV CP-specific antibodies (Figure 4a, IB: anti-CP panel). As expected, no BaMV CP was detected in the protein extracts from mock-inoculated leaves (Figure 4a, IB: anti-CP panel lane 2). BVP1 and BVP1-9 (Figure 4a, IB: anti-CP panel lane 4 and 5) were both detected in the protein extracts of leaves inoculated with pBVP1 or pBVP1-9, which migrated slightly faster than the chimeric CP from pBVP1-7A7-, pBVP1-9A9-, pBVP1-7A9-, pBVP1-9A7-, pBVP1-7AG9-, and pBVP1-9AG7-inoculated leaves (Figure 4a, IB: anti-CP panel lane 6 to 11). In contrast, the BaMV CP signal was lower in the pBVP1-9A7A7-inoculated leaves (Figure 4a, IB: anti-CP panel lane 12 and 14). Western blotting analysis using FMDV VP1-specific antiserum was performed to further verify that the chimeric CP generated from pBVP1s-inoculated leaves harbored FMDV VP1 epitopes (Figure 4a, IB: anti-VP1 panel, IB: anti-VP1-97 panel, IB: anti-VP1-99 panel). No protein band was detected using the VP1-97-specific antiserum in pBS-d35CP, pBVP1-9 or pBVP1-9A9-inoculated leaf protein extracts (Figure 4a, IB: anti-VP1-97 panel). The protein extracts from pBS-d35CP, pBVP1 or pBVP1-7A7-inoculated leaves using VP1-99-specific antiserum showed similar results, with no protein band detected (Figure 4a, IB: anti-VP1-99 panel).

Inconsistent results were obtained when total protein extracts from infected *N. benthamiana* systemic leaves were assayed using Western blotting. No protein band was detected by the BaMV CP- or VP1-specific antiserum in protein extracts from systemic leaves inoculated with pBVP1-7A7, pBVP1-9A9, pBVP1-7A9, pBVP1-9A7, pBVP1-7AG9, pBVP1-9AG7, or pBVP1-9A7A7 (Figure 4b, IB: anti-CP panel, IB: anti-VP1 panel, IB: anti-VP1-97 panel, IB: anti-VP1-99 panel). Therefore, these results suggest that FMDV VP1 epitope fragments (77 and 82 amino acids long) did not affect the infection but affected the systemic movement of the chimeric viruses.

### 3.6. Accumulation of the Chimeras with Heterologus Peptides

To determine whether the accumulation levels of BVP1s chimeric viruses were produced normally in *C. quinoa*, total protein from the inoculated leaves infected with H_2_O (mock), pBS-d35CP, pBVP1, pBVP1-9, pBVP1-7A7, pBVP1-9A9, pBVP1-7A9, pBVP1-9A7, pBVP1-7AG9, pBVP1-9AG7, or pBVP1-9A7A7 were analyzed using ELISA, and the results were categorized into six main groups as previously described (Table 1). It was found that chimeric BVP1, BVP1-9, and BS-d35CP had similar accumulation levels (Table 1). However, the accumulation level of the BVP1-7A7 virus was significantly higher than that of the BVP1-9A9 virus (Table 1). In addition, BVP1-7A9 and BVP1-9A7 viruses had the same pI value, charge, and length of foreign fragments when fused, but the chimeric BVP1-9A7 virus had a relatively lower accumulation level compared to the BVP1-7A9 virus (Table 1). Similar accumulation results were observed for the BVP1-7AG9 and BVP1-9AG7 viruses, respectively (Table 1).

Furthermore, the chimeric BVP1-7A9, BVP1-7AG9, and chimeric BVP1-9A7 and BVP1-9AG7 viruses had similar pI values and charges but different spacers. The chimeric BVP1-7A9 virus had a relatively lower accumulation level compared to BVP1-7AG9 and BVP1-9A7, and BVP1-9AG7 viruses also showed similar results (Table 1). While the insertion and expression of large foreign FMDV VP1 antigens (77 or 82 amino acids long) did not interfere with replication, the characteristics of large foreign N-terminus fragments had a greater impact on the accumulation of chimeras than the characteristics of the C-terminus fragments. Additionally, using different spacers may improve the structural barriers of foreign proteins and thus increase chimera accumulation.

### 3.7. Morphology of the Chimeras Particles with Heterologus Peptides

**Figure 5:**
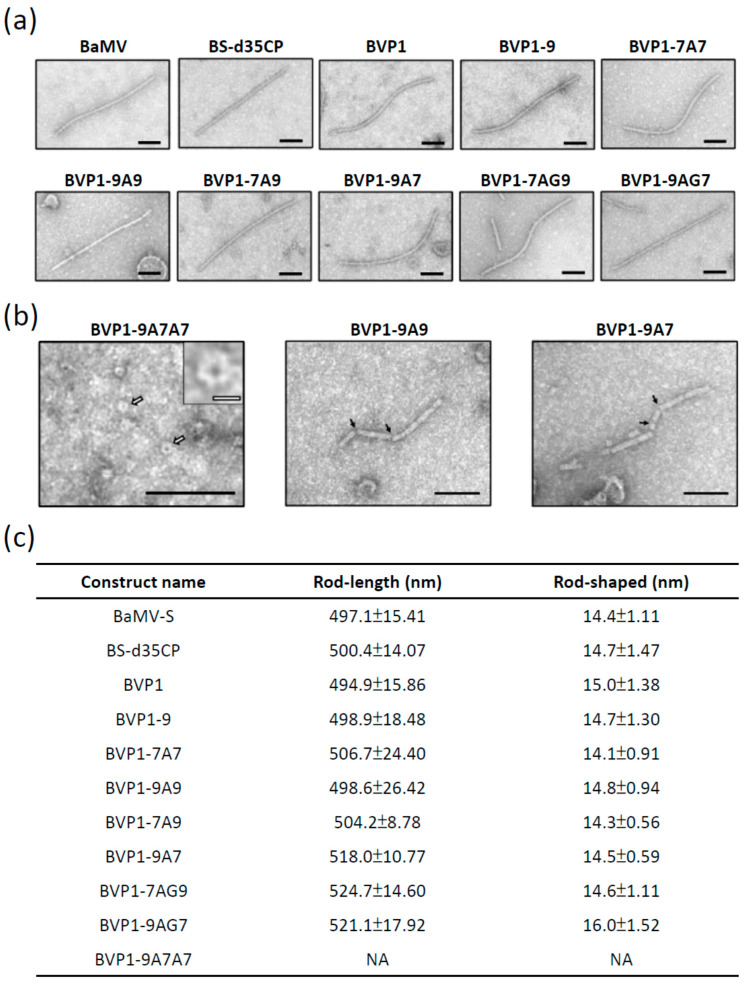
Transmission electron micrographs of negatively stained (UAc) BVP1s (various FMDV VP1 epitopes expressed on BaMV) particles. Serologically specific electron microscopy (SSEM) micrographs of BVP1s CVPs extracted from *C. quinoa* leaves inoculated with the chimeras indicated below the photographs. (**a**) Complete BVP1s virus particles, and (**b**) incomplete or abnormal BVP1s virus particles were detected by SSEM indexing using BaMV antiserum and subjected to examination by transmission electron microscopy. The magnification levels were 96,000× for BVP1-9A7A7 and 46,000× for the other samples. Inset BVP1-9A7A7 picture shows the same sample at higher magnification. White arrows represent disks and black arrows represent break points. The black scale bars represent 100 nm, and the white scale bar represents 10 nm. (**c**) The diameters and lengths of the corresponding virus particles. Numbers represent the mean diameters and lengths, respectively, calculated from 20 independent samples with standard deviation indicated. NA indicates not applicable.

Following the successful observation of chimeric BVP1s CP expression in inoculated plants, it was important to examine the extent of CVP self-assembly. To investigate BVP1s CVPs formed by infected chimeras in plants, crude sap from inoculated *C. quinoa* leaves was analyzed using SSEM. SSEM confirmed that the large FMDV VP1 antigen (77 or 82 amino acids) fused to the N-terminus of BaMV CP did not affect its ability to self-assemble into CVPs, and that BVP1-7A7, BVP1-9A9, BVP1-7A9, BVP1-9A7, BVP1-7AG9, and BVP1-9AG7 CVPs appeared to be typical flexuous rod-shaped, similar to the wild-type BaMV virions (Figure 5a).

However, the chimera BVP1-9A7A7 (fused of FMDV VP1 epitopes 117 amino acids) was observed as incomplete virus particles or disks, as shown in Figure 5b (left panel). Furthermore, BVP1-9A9 and BVP1-9A7 chimeras were also observed to be partially unstable or incomplete virus particles (Figure 5b middle panel, right panel). Meanwhile, a majority of the BVP1s CVPs appeared to be rod-shaped, similar to the wild-type BaMV particles. The average diameter of the BVP1s particles was approximately 15 nm, with an average length of ~500 nm, similar to that of the wild-type BaMV virus (Figure 5c). No significant difference was observed between BVP1s CVPs and the different BVP1s constructs (Figure 5c). Therefore, these results suggest that the large FMDV VP1 antigens (77 or 82 amino acids) fused to chimeric BVP1s can self-assemble into typical rod-shaped BaMV CVPs. However, although chimeric CPs directly fused with large foreign protein can self-fold into incomplete virus particles or disks, it still affects their assembly into complete CVPs.

### 3.8. Chimeras with Heterologus Peptides Are Stable during Successive Passages in Plants

**Figure 6:**
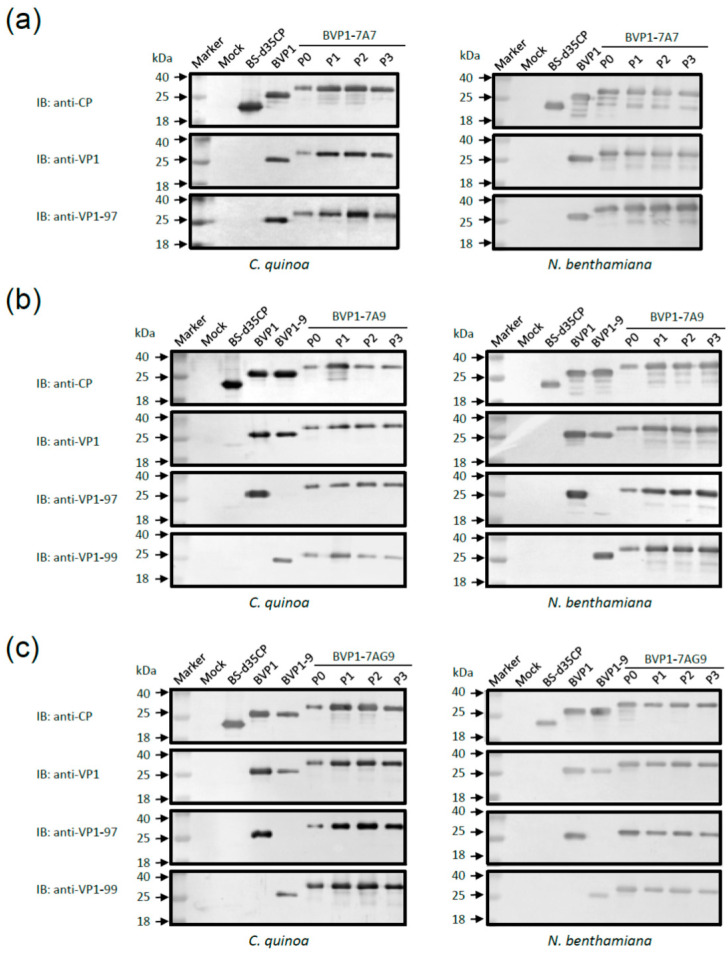
Stability analysis of the chimeric BVP1s over sequential passages in *C. quinoa* or *N. benthamiana* plants by SDS-PAGE immunoblot. *C. quinoa* (left panel) or *N. benthamiana* leaves (right panel) were inoculated with H_2_O (mock) or with recombinant plasmids pBS-d35CP, pBVP1, pBVP1-9, pBVP1-7A7, pBVP1-7A9, or pBVP1-7AG9, respectively. BVP1s P0 represents the initial inoculation with the plasmid DNA as inoculums, whereas P1 to P3 stands for the first to third passage using crude leaf sap from P0 as inocula, respectively. Total proteins extracted from leaves inoculated with pBVP1-7A7 (**a**), pBVP1-7A9 (**b**), or pBVP1-7AG9 (**c**) leaves were separated using 12% SDS-PAGE. The proteins were transferred to PVDF membranes and reacted with antisera against BaMV CP (anti-CP), FMDV VP1 (anti-VP1), FMDV VP1 (O/TAW/97 specificity, anti-VP1-97), or FMDV VP1 (O/TAW/99 specificity, anti-VP1-99), respectively. The relative molecular weights (in kDa) are given on the left of each panel. IB, immunoblot.

To examine the stability of the chimeric BVP1s virus during successive passages in plants, infectious recombinant pBVP1-7A7, pBVP1-7A9, and pBVP1-7AG9 plasmids were inoculated onto *C. quinoa* or *N. benthamiana* leaves to generate P0 inoculants. The inoculants were then subjected to three sequential transmissions (P1–P3) in *C. quinoa* and *N. benthamiana* (Figure 6). The chimeric BVP1s CPs were monitored at each transfer via Western blot analysis using the corresponding antisera. All inoculated leaves developed lesions, symptoms and numbers similar to those observed in P0-infected plants. After three serial passages in *C. quinoa* and *N.benthamiana*, the results of Western blot analysis using antisera specific to BaMV CP or FMDV VP1 clearly confirmed the stability of chimeric BVP1s infection, and still continued to express FMDV antigens (Figure 6). Additionally, the chimeric BVP1-9A7A7 virus can infect plants but is not stable for sequential transmission infection. However, the results of these serial passages demonstrated that the insertion of larger FMDV VP1 antigens fused to BaMV CP may still be partially unstable in the chimeric BVP1s virus.

## 4. Discussion

Here, we showed that the restrictions on designing filamentous CVPs with increased fragment length directly expressing foreign proteins may help develop vaccines using the plant CVP platform. The multitude of viruses and engineered vectors provide diverse virion stability, capacity for foreign gene expression and ability to induce effective immune responses, such as tobacco mosaic virus (TMV) [38,39], PVX [40], cucumber green mottle mosaic virus [41], BaMV [9,11,12] and pepino mosaic virus [42]. These viruses are a useful resource for vaccine production.

This study aimed to investigate the BaMV CVPs and BaMV CP N-terminus, as PVX was predicted to be exposed at the virion surface [22,43] and may be crucial for CVP morphology, stability and infectivity [18]. In the present study, our results showed that hydrophobic foreign proteins exposed to the CVP surface affected the infection and accumulation of chimeric BaMV (Table S1, Figure 2). The PVX virion surfaces are usually hydrophilic, with a deeply grooved surface where bound water molecules help maintain the virion surface structure [32,34]. This hydrophobic effect is important for protein folding [44], and minimizing the number of hydrophobic side chains exposed to water is the principal driving force behind the folding process [45]. Therefore, the hydrophilic amino acids on the BaMV CVP surface are important for maintaining the infection and accumulation stability of the chimeric virus.

The filamentous PVX virion contains approximately 1300 identical CP subunits [46], and most or all flexible filamentous plant viruses are structurally related [22,47]. Individual filamentous virus CP subunits first self-assemble into helical disks and further assemble along the central RNA into full-size virions [48]. Although BaMV has a flexible N-terminal extension that allows deformation but still maintains structural integrity [22], in this study we found that foreign peptides fused on the BaMV CP N-terminal contain more helix and strand secondary structures or C-terminus-containing complex strands, which affect the infection and accumulation of the of chimeric virus (Table S1, Figure 3). The secondary structure of proteins is important for protein folding and stability, and these effects may aid the correct folding of chimeric CP subunits. In addition, the cysteine residues in the foreign peptides are directly related to changes in TMV particle yield, morphology and stability [19]. This study also found that when the foreign proteins contain cysteine, thjis may cause changes in the original CP disulfide bond, which then leads to the CP folding incorrectly and unable to assemble into the correct CVP, resulting in a decrease in the accumulation of chimeras (Table S1). Consequently, the amino acid sequences or secondary structure of foreign proteins directly fused onto the CP may affect the folding and self-assembly of chimeric viral CP subunits, thereby reducing recombinant virus accumulation.

A limitation of CVPs is virus assembly failure, when large amino acid length peptides are incorporated into the CP. Only peptides less than 25 amino acids were successfully introduced into the TMV CP [14]. Moreover, large fragments from foreign proteins affected chimeric CPMV accumulation, assembly and movement [20]. Chimeric CPMV particles with insertions greater than 30 amino acids have impaired systemic spread rates and a range of deletions in virus particles isolated from the upper leaves [20]. These observations indicate that insertions greater than a certain length are deleterious to the viral replication cycle. Therefore, improving the performance of plant viral vectors with longer foreign proteins is important. Because the N-terminal amino acid of BaMV CP is exposed [22], a part of CP removal was chosen, mainly because it was hypothetically more suitable for longer peptide displays than the wild-type virus, but also because it is putatively less immunogenic due to its reduced dimensions [16,49]. It has been confirmed that the formation of functional BaMV virus particles is not affected significantly by the removal of CP N-terminal amino acids [9,23], as in PVX (Figure S3, Figure S4). Using this CP N-terminal amino acid removal virus vector, it was found that in small foreign protein fragments (less than 10 amino acids), these factors could weakly affect the accumulation of recombinant viruses (Figure 2, Table S1). When the foreign protein fragments were longer, the influence of recombinant virus accumulation was more evident (Figure 2, Table S1).

Previous studies found that the flexibility of foreign proteins may also affect the recombinant virus. Linkers have been shown to have an increasingly important role in the construction of stable bioactive fusion proteins. Linkers can connect proteins together, improve the production of fusion proteins and increase expression yield [50]. Interestingly, by linking the two antigens with the flexible (AAA or AAAGGGGS) spacer, we also found longer foreign fragments (77 or 82 amino acids) that were FMDV VP1 epitopes, the expression of which formed recombinant virus particle packages (Figure 6). However, the longest foreign peptide (117 amino acids) may affect the assembly of chimeric disks into filamentous virions (Figure 6). Additionally, longer foreign protein fragments have a more obvious impact on the systemic movement of recombinant viruses (Figure 4). Therefore, the length of the foreign protein may affect the folding of the chimeric viral CP subunits, which can subsequently affect CVP assembly and accumulation.

Mutual restrictions between virus and host interactions [20,51,52] could affect the ability of the chimeric virus to spread systemically throughout the entire plant. An example is the chimeric Turnip mosaic virus, for which inserted sequences and host plants affect both its stability and expression levels [51]. The chimeric PVX virus may induce severe symptomatology and, owing to the interference of the modified CP with chloroplast functionality, lead to the rapid disruption of these organelles [53]. Therefore, recombinant viruses resulting from foreign protein fragments containing special functional sequences with the host can have accumulative effects on virus replication. The arginyl–glycyl–aspartic acid (RGD) motif, an attachment site on the host cell membrane, is a potential therapeutic agent for the treatment of diseases such as thrombosis and cancer [54]. The RGD motif has also been shown in FMDV [55,56] and flaviviruses [57] to be involved in cell attachment, infection initiation and virus binding to putative receptors. Previous studies have shown that FMDV epitopes containing an RGD motif can potentially cause recombinant CPMV to bind to cell membranes, thus blocking the spread of chimeras and decreasing the difficulty of isolating of progeny virus particles [58]. Additionally, recombinant TMV constructs expressing fragments containing an RGD motif resulted in a lower purity of recombinant viral particles collected from infected tobacco plants [19]. In this study, peptide JC had a cell attachment sequence RGD motif. The peptide JB, with an Arg-to-Met change in the peptide JC sequence, mutated the RGD motif (Table S1). Plants inoculated with these two chimeras showed that the loss of the RGD motif may increase the accumulation of the chimeric virus (Table S1). Therefore, foreign proteins containing signals that control interactions in hosts can affect CVP accumulation.

From the results of the current study, we can conclude that the N-terminal of CP plays a pivotal role in the morphology, stability and accumulation of most flexible filamentous plant viruses. This study found that the N-terminal hydrophilic portion is an important factor in virion stability and causes changes in the chimeric virus accumulation level. Another important factor is the CP secondary structure, which changes to a disulfide bond. Other important factors include the length of the inserted foreign peptide, the pI value and charge of the inserted foreign peptide N-terminus, and the presence of foreign proteins containing the signal for host interactions. These factors have different effects on chimeric virus accumulation. Furthermore, adding suitable flexible spacers with foreign proteins can effectively improve chimeric virus accumulation. These findings indicate that peptide display technology, aimed at the mass production of pharmaceutically interesting heterologous sequences in plants, may also be a useful tool to unravel the complexity of plant-virus interactions during the infection cycle.

## 5. Conclusions

To our knowledge, this is the first report to demonstrate CVPs with increased fragment length that directly express foreign proteins. The limited ability of plant virus-based vectors to express longer foreign peptides on CVPs has negatively impacted their utility for applications requiring longer peptides. This is also an important production limitation for the importance of various vaccines using plant CVPs. Therefore, this study provides new insights into effective and useful CVP-based vaccine candidate production in animals and humans.

## Figures and Tables

**Table 1:** List of the N-terminal heterologus peptides of the chimeric BaMV CP putatively encoded by each construct.

Peptide Name	Amino Acid Sequence ^†^	Construct Name	Peptide Length	Peptide Charge	Peptide pI Value ^‡^	Accumulation ^§^
F-E	TVYNGSSKYGDTSTNNVRGDLQVLAQKAERTLPTSFN	pBVP1	37	+1	8.10	V
F-F	TVYNGNCKYGESPVTNVRGDLQVLAQKAARTLPTSFN	pBVP1-9	37	+2	9.10	V
F-EE	TVYNGSSKYGDTSTNNVRGDLQVLAQKAERTLPTSFNAAATVYNGSSKYGDTSTNNVRGDLQVLAQKAERTLPTSFN	pBVP1-7A7	77	+2	9.15	IV
F-FF	TVYNGNCKYGESPVTNVRGDLQVLAQKAARTLPTSFNAAATVYNGNCKYGESPVTNVRGDLQVLAQKAARTLPTSFN	pBVP1-9A9	77	+4	9.39	I
F-EF	TVYNGSSKYGDTSTNNVRGDLQVLAQKAERTLPTSFNAAATVYNGNCKYGESPVTNVRGDLQVLAQKAARTLPTSFN	pBVP1-7A9	77	+3	9.30	II
F-FE	TVYNGNCKYGESPVTNVRGDLQVLAQKAARTLPTSFNAAATVYNGSSKYGDTSTNNVRGDLQVLAQKAERTLPTSFN	pBVP1-9A7	77	+3	9.30	I
F-EF1	TVYNGSSKYGDTSTNNVRGDLQVLAQKAERTLPTSFNAAASGGGGTVYNGNCKYGESPVTNVRGDLQVLAQKAARTLPTSFN	pBVP1-7AG9	82	+3	9.30	III
F-FE1	TVYNGNCKYGESPVTNVRGDLQVLAQKAARTLPTSFNAAASGGGGTVYNGSSKYGDTSTNNVRGDLQVLAQKAERTLPTSFN	pBVP1-9AG7	82	+3	9.30	II
F-FEE	TVYNGNCKYGESPVTNVRGDLQVLAQKAARTLPTSFNAAATVYNGSSKYGDTSTNNVRGDLQVLAQKAERTLPTSFNAAATVYNGSSKYGDTSTNNVRGDLQVLAQKAERTLPTSFN	pBVP1-9A7A7	117	+4	9.37	I/0

^†^ Word subscript line indicate linker fragment; ^‡^ isoelectric point; ^§^ accumulation of chimeric BaMV virus in *C. quinoa* was normalized BS-d35CP (100%) to account for chimeric BaMV virus 0: no-infection, I: 1–20%, II: 21–40%, III: 41–60%, IV: 61–80%, V: 81–100%. Spacer sequences are underlined.

## Data Availability

The data presented in this study are available on request from the corresponding author.

## Supplementary Materials

The following are available online at https://www.mdpi.com/article/10.3390/v13040572/s1, Methods S1. Construction of chimeric BaMV infectious clones. Methods S2. PVX chimeric particles produces antibodies for the distinction between FMDV O strain topotypes. Figure S1. Comparison of the nucleotide and amino acid sequences of the PVX coat protein (CP) (GenBank ID: AAA03493.1) and PVX Taiwan strain (PVX-T) CP (GenBank ID: AF272736.2); Figure S2. Comparison of the amino acid sequences and secondary structure of the PVX Taiwan strain (PVX-T) coat protein (CP) (GenBank ID: AF272736.2) and BS-d35CP; Figure S3. Schematic representation of the recombinant pPVP1-97s and pPVP1-99s based on PVX genome; Figure S4. Symptoms and molecular analysis of plants inoculated with the potato virus X viral expression vectors; Figure S5. PVX chimeric particles produce antibodies for the distinction between FMDV O strain topotypes. Table S1. List of the nonconsensus N-terminal sequences of the chimeric BaMV CP putatively encoded by each construct. Table S2. The primers used in this study.

## References

[1] Rybicki E.P. (2010). Plant-made vaccines for humans and animals. Plant. Biotechnol. J..

[2] Abrahamian P., Hammond R.W., Hammond J. (2020). Plant Virus-Derived Vectors: Applications in Agricultural and Medical Biotechnology. Annu. Rev. Virol..

[3] Balke I., Zeltins A. (2020). Recent Advances in the Use of Plant Virus-Like Particles as Vaccines. Viruses.

[4] Ruiz V., Mozgovoj M.V., Dus Santos M.J., Wigdorovitz A. (2015). Plant-produced viral bovine vaccines: What happened during the last 10 years?. Plant. Biotechnol. J..

[5] Lei X., Cai X., Yang Y. (2020). Genetic engineering strategies for construction of multivalent chimeric VLPs vaccines. Expert Rev. Vaccines.

[6] Gerloni M., Xiong S., Mukerjee S., Schoenberger S.P., Croft M., Zanetti M. (2000). Functional cooperation between T helper cell determinants. Proc. Natl. Acad. Sci. USA.

[7] Smith M.L., Lindbo J.A., Dillard-Telm S., Brosio P.M., Lasnik A.B., McCormick A.A., Nguyen L.V., Palmer K.E. (2006). Modified tobacco mosaic virus particles as scaffolds for display of protein antigens for vaccine applications. Virology.

[8] Wang Z.B., Xu J. (2020). Better Adjuvants for Better Vaccines: Progress in Adjuvant Delivery Systems, Modifications, and Adjuvant-Antigen Codelivery. Vaccines.

[9] Yang C.D., Liao J.T., Lai C.Y., Jong M.H., Liang C.M., Lin Y.L., Lin N.S., Hsu Y.H., Liang S.M. (2007). Induction of protective immunity in swine by recombinant bamboo mosaic virus expressing foot-and-mouth disease virus epitopes. BMC Biotechnol..

[10] Liew P.S., Hair-Bejo M. (2015). Farming of Plant-Based Veterinary Vaccines and Their Applications for Disease Prevention in Animals. Adv. Virol..

[11] Chen T.H., Chen T.H., Hu C.C., Liao J.T., Lee C.W., Liao J.W., Lin M.Y., Liu H.J., Wang M.Y., Lin N.S. (2012). Induction of protective immunity in chickens immunized with plant-made chimeric Bamboo mosaic virus particles expressing very virulent Infectious bursal disease virus antigen. Virus Res..

[12] Chen T.H., Hu C.C., Liao J.T., Lee Y.L., Huang Y.W., Lin N.S., Lin Y.L., Hsu Y.H. (2017). Production of Japanese Encephalitis Virus Antigens in Plants Using Bamboo Mosaic Virus-Based Vector. Front. Microbiol..

[13] Lico C., Chen Q., Santi L. (2008). Viral vectors for production of recombinant proteins in plants. J. Cell Physiol..

[14] Jiang L., Li Q., Li M., Zhou Z., Wu L., Fan J., Zhang Q., Zhu H., Xu Z. (2006). A modified TMV-based vector facilitates the expression of longer foreign epitopes in tobacco. Vaccine.

[15] Bendahmane M., Koo M., Karrer E., Beachy R.N. (1999). Display of epitopes on the surface of tobacco mosaic virus: Impact of charge and isoelectric point of the epitope on virus-host interactions. J. Mol. Biol..

[16] Lico C., Capuano F., Renzone G., Donini M., Marusic C., Scaloni A., Benvenuto E., Baschieri S. (2006). Peptide display on Potato virus X: Molecular features of the coat protein-fused peptide affecting cell-to-cell and phloem movement of chimeric virus particles. J. Gen. Virol..

[17] Uhde-Holzem K., Fischer R., Commandeur U. (2007). Genetic stability of recombinant potato virus X virus vectors presenting foreign epitopes. Arch. Virol..

[18] Betti C., Lico C., Maffi D., D’Angeli S., Altamura M.M., Benvenuto E., Faoro F., Baschieri S. (2012). Potato virus X movement in Nicotiana benthamiana: New details revealed by chimeric coat protein variants. Mol. Plant. Pathol..

[19] Li Q., Jiang L., Li M., Li P., Zhang Q., Song R., Xu Z. (2007). Morphology and stability changes of recombinant TMV particles caused by a cysteine residue in the foreign peptide fused to the coat protein. J. Virol. Methods.

[20] Porta C., Spall V.E., Findlay K.C., Gergerich R.C., Farrance C.E., Lomonossoff G.P. (2003). Cowpea mosaic virus-based chimaeras. Effects of inserted peptides on the phenotype, host range, and transmissibility of the modified viruses. Virology.

[21] Canizares M.C., Nicholson L., Lomonossoff G.P. (2005). Use of viral vectors for vaccine production in plants. Immunol. Cell Biol..

[22] DiMaio F., Chen C.C., Yu X., Frenz B., Hsu Y.H., Lin N.S., Egelman E.H. (2015). The molecular basis for flexibility in the flexible filamentous plant viruses. Nat. Struct. Mol. Biol..

[23] Lan P., Yeh W.B., Tsai C.W., Lin N.S. (2010). A unique glycine-rich motif at the N-terminal region of Bamboo mosaic virus coat protein is required for symptom expression. Mol. Plant. Microbe Interact..

[24] Muthamilselvan T., Lee C.W., Cho Y.H., Wu F.C., Hu C.C., Liang Y.C., Lin N.S., Hsu Y.H. (2016). A transgenic plant cell-suspension system for expression of epitopes on chimeric Bamboo mosaic virus particles. Plant. Biotechnol. J..

[25] Ferre F., Clote P. (2006). DiANNA 1.1: An extension of the DiANNA web server for ternary cysteine classification. Nucleic Acids Res..

[26] McGuffin L.J., Bryson K., Jones D.T. (2000). The PSIPRED protein structure prediction server. Bioinformatics.

[27] Lin M.K., Chang B.Y., Liao J.T., Lin N.S., Hsu Y.H. (2004). Arg-16 and Arg-21 in the N-terminal region of the triple-gene-block protein 1 of Bamboo mosaic virus are essential for virus movement. J. Gen. Virol..

[28] Lin N.S., Chen C.C. (1991). Association of bamboo mosaic virus (BaMV) and BaMV-specific electron-dense crystalline bodies with chloroplasts. Phytopathology.

[29] Wang J.H., Liang C.M., Peng J.M., Shieh J.J., Jong M.H., Lin Y.L., Sieber M., Liang S.M. (2003). Induction of immunity in swine by purified recombinant VP1 of foot-and-mouth disease virus. Vaccine.

[30] Lin N.S. (1984). Gold-IgG complexes improve the detection and identification of viruses in leaf dip preparations. J. Virol. Methods.

[31] Rose G.D., Wolfenden R. (1993). Hydrogen bonding, hydrophobicity, packing, and protein folding. Annu. Rev. Biophys. Biomol. Struct..

[32] Baratova L.A., Fedorova N.V., Dobrov E.N., Lukashina E.V., Kharlanov A.N., Nasonov V.V., Serebryakova M.V., Kozlovsky S.V., Zayakina O.V., Rodionova N.P. (2004). N-Terminal segment of potato virus X coat protein subunits is glycosylated and mediates formation of a bound water shell on the virion surface. Eur. J. Biochem..

[33] Gasteiger E., Hoogland C., Gattiker A., Duvaud S., Wilkins M.R., Appel R.D., Bairoch A., Walker J.M. (2005). Protein Identification and Analysis Tools on the ExPASy Server. The Proteomics Protocols Handbook.

[34] Grinzato A., Kandiah E., Lico C., Betti C., Baschieri S., Zanotti G. (2020). Atomic structure of potato virus X, the prototype of the Alphaflexiviridae family. Nat. Chem. Biol..

[35] Anthony R.P., Paredes A.M., Brown D.T. (1992). Disulfide bonds are essential for the stability of the Sindbis virus envelope. Virology.

[36] Krondiris J.V., Sideris D.C. (2002). Intramolecular disulfide bonding is essential for betanodavirus coat protein conformation. J. Gen. Virol..

[37] Trinh R., Gurbaxani B., Morrison S.L., Seyfzadeh M. (2004). Optimization of codon pair use within the (GGGGS)3 linker sequence results in enhanced protein expression. Mol. Immunol..

[38] Donson J., Kearney C.M., Hilf M.E., Dawson W.O. (1991). Systemic expression of a bacterial gene by a tobacco mosaic virus-based vector. Proc. Natl. Acad. Sci. USA.

[39] Turpen T.H., Reinl S.J., Charoenvit Y., Hoffman S.L., Fallarme V., Grill L.K. (1995). Malarial epitopes expressed on the surface of recombinant tobacco mosaic virus. Biotechnology (N Y).

[40] Chapman S., Kavanagh T., Baulcombe D. (1992). Potato virus X as a vector for gene expression in plants. Plant J..

[41] Ooi A., Tan S., Mohamed R., Rahman N.A., Othman R.Y. (2006). The full-length clone of cucumber green mottle mosaic virus and its application as an expression system for Hepatitis B surface antigen. J. Biotechnol..

[42] Sempere R.N., Gomez P., Truniger V., Aranda M.A. (2011). Development of expression vectors based on pepino mosaic virus. Plant Methods.

[43] Nemykh M.A., Efimov A.V., Novikov V.K., Orlov V.N., Arutyunyan A.M., Drachev V.A., Lukashina E.V., Baratova L.A., Dobrov E.N. (2008). One more probable structural transition in potato virus X virions and a revised model of the virus coat protein structure. Virology.

[44] Dill K.A., Truskett T.M., Vlachy V., Hribar-Lee B. (2005). Modeling water, the hydrophobic effect, and ion solvation. Annu Rev. Biophys. Biomol. Struct..

[45] Pace C.N., Shirley B.A., McNutt M., Gajiwala K. (1996). Forces contributing to the conformational stability of proteins. FASEB J..

[46] Parker L., Kendall A., Stubbs G. (2002). Surface features of potato virus X from fiber diffraction. Virology.

[47] Kendall A., McDonald M., Bian W., Bowles T., Baumgarten S.C., Shi J., Stewart P.L., Bullitt E., Gore D., Irving T.C. (2008). Structure of flexible filamentous plant viruses. J. Virol..

[48] Tremblay M.H., Majeau N., Gagne M.E., Lecours K., Morin H., Duvignaud J.B., Bolduc M., Chouinard N., Pare C., Gagne S. (2006). Effect of mutations K97A and E128A on RNA binding and self assembly of papaya mosaic potexvirus coat protein. FEBS J..

[49] Koenig R., Torrance L. (1986). Antigenic analysis of potato virus X by means of monoclonal antobodies. J. Gen. Virol..

[50] Chen X., Zaro J.L., Shen W.C. (2013). Fusion protein linkers: Property, design and functionality. Adv. Drug Deliv. Rev..

[51] Chen C.C., Chen T.C., Raja J.A., Chang C.A., Chen L.W., Lin S.S., Yeh S.D. (2007). Effectiveness and stability of heterologous proteins expressed in plants by Turnip mosaic virus vector at five different insertion sites. Virus Res..

[52] Ahlquist P., Schwartz M., Chen J., Kushner D., Hao L., Dye B.T. (2005). Viral and host determinants of RNA virus vector replication and expression. Vaccine.

[53] Betti C., Lico C., Iriti M., D’Angeli S., Benvenuto E., Baschieri S., Faoro F. (2010). A chimeric Potato virus X encoding a heterologous peptide affects Nicotiana benthamiana chloroplast structure. Plant. Biosyst..

[54] D’Souza S.E., Ginsberg M.H., Plow E.F. (1991). Arginyl-glycyl-aspartic acid (RGD): A cell adhesion motif. Trends Biochem. Sci..

[55] Fox G., Parry N.R., Barnett P.V., McGinn B., Rowlands D.J., Brown F. (1989). The cell attachment site on foot-and-mouth disease virus includes the amino acid sequence RGD (arginine-glycine-aspartic acid). J. Gen. Virol..

[56] Mason P.W., Rieder E., Baxt B. (1994). RGD sequence of foot-and-mouth disease virus is essential for infecting cells via the natural receptor but can be bypassed by an antibody-dependent enhancement pathway. Proc. Natl. Acad. Sci. USA.

[57] Lobigs M., Usha R., Nestorowicz A., Marshall I.D., Weir R.C., Dalgarno L. (1990). Host cell selection of Murray Valley encephalitis virus variants altered at an RGD sequence in the envelope protein and in mouse virulence. Virology.

[58] Porta C., Spall V.E., Loveland J., Johnson J.E., Barker P.J., Lomonossoff G.P. (1994). Development of cowpea mosaic virus as a high-yielding system for the presentation of foreign peptides. Virology.

