# Supplementary material for: Stable Display of Artificially Long Foreign Antigens on Chimeric Bamboo mosaic virus Particles
Source: Viruses. 2021 Mar 29;13(4):572. doi: 10.3390/v13040572 (PMC8067224; doi:10.3390/v13040572)
Supplement: Supplementary file 1 [file viruses-13-00572-s001.pdf]

Supplementary Material

Stable Display of Artificially Long Foreign Antigens on Chimeric *Bamboo mosaic virus* Particles

Tsung-Hsien Chen, Chung-Chi Hu, Chin-Wei Lee, Yu-Min Feng, Na-Sheng Lin and Yau-Heiu Hsu

Table S1. List of the non-consensus N-terminal sequences of the chimeric BaMV CP putatively encoded by each construct.

Peptide name	Amino acid sequence	Construct name	peptide length	peptide charge	peptide pI value†	Hydrophilicity value‡	Accumulation§
I-A	MSLLTEVETPIRNEWGCRCNGSSD	pBIA11	24	-2	4.41	0.392	III
I-B	MSLLTEVETPTRSEWECRCSDSSD	pBIA1109	24	-4	4.22	1.000	II
I-C	MSLLTEVETPIRNEWGCRCNDSSD	pBIA32	24	-3	4.18	0.632	II
I-D	MSLLTEVETPIRNEWGSRSNDSSD	pBIA32S24	24	-3	4.18	0.840	V
I-E	MSLLTEVEPIRNEWGSRSNDSSDPSR	pBIA32S27	27	-2	4.51	0.986	V
I-F	MSLLTEVETPTRNEWECRCSDSSD	pBIA51	24	-4	4.08	0.992	II
I-G	MSLLTEVETPTRNEWECKCIDSSD	pBIA51I	24	-4	4.08	0.824	V
I-H	CNTKCQTP	pBHA8	8	+1	8.06	0.133	V
I-I	IDGWYGYHHQN	pBHA21	11	-1	5.97	-1.233	I
I-J	QKSTQNAIDGITNKVNSVI	pBHA22	19	+1	8.59	0.009	II
I-M	HEASSGVSSACPYQGKSSFFRNVVWLIKK	pBIAHA1	29	+3	9.63	-0.273	0
I-N	GVSSACPYQGKSSFFRNVVWLIKK	pBIAHA2	24	+4	10.03	-0.536	0
I-O	SSACPYQGKSSFFRNVVWLIKK	pBIAHA3	22	+4	10.03	-0.452	0
I-P	ACPYQGKSSFFRNVVWLIKK	pBIAHA4	20	+4	10.03	-0.552	0
I-Q	PYQGKSSFFRNVVWLIKK	pBIAHA5	18	+4	10.47	-0.453	0
I-R	KSSFFRNVVWLIKK	pBIAHA6	14	+4	11.26	-0.293	0
I-S	SSACPYQGKSSFFRNVVWLI	pBIAHA7	20	+2	9.30	-1.067	0
I-T	ACPYQGKSSFFRNVVWLI	pBIAHA8	18	+2	9.32	-1.242	0
F-A	SSKYGDTSTN	pBVP197m	10	0	5.55	0.727	V
F-B	NCKYGESPVT	pBVP199m	10	0	5.99	0.236	V
F-C	TSKYSAGGTG	pBVP1Am	10	+1	8.26	0.000	IV
F-D	KTTYGETTRR	pBVP1Asm	10	+2	9.99	1.473	V
J-A	DKLALKGTTYGMCTEKFSFAKNPADTGHGT	pBJ1-30	30	+1	8.15	0.277	III
J-B	GDSYIVVGMGDKQINHHWHKAGST	pBJ88-111	24	0	7.02	-0.240	III
J-C	GDSYIVVGRGDKQINHHWHKAGST	pBJ88-111R	24	0	8.52	0.104	II
L-A	MKRGLCCVLLLCGAVFVS	pBS-Td35CP	18	+2	8.68	-1.337	0
L-B	AKFVAAWTLKAAA	pBS-Pd35CP	13	+2	10	-0.943	I
L-C	CDCRGDCFC	pBS-Rd35CP	9	-1	4.21	-0.060	II

† Isoelectric point; ‡ According to Hopp and Woods (1981); §Accumulation of chimeric BaMV virus in *C. quinoa* was normalized BS-d35CP (100%) to account for chimeric BaMV virus 0: no-infection, I: 1%–20%, II: 21%–40%, III: 41%–60%, IV: 61%–80%, V: 81%–100%.

Methods S1. Construction of chimeric BaMV - infectious clones.

The DNA fragment coding for the seasonal influenza A (A/hvPR8/34/H1N1) M2e (MSLLTEVETPIRNEWGCRCNGSSD, IA) was amplified by PCR with primers p11F and p11R (Table S2). The DNA fragment coding for the swine influenza viruses (A/Taiwan/T1821/2009/H1N1) M2e (MSLLTEVETPTRSEWECRCSDSSD, IB) was amplified by PCR with primers p1109F and p1109R (Table S2). The DNA fragment coding for the seasonal influenza A (A/Western Australia/78/2005/H3N2) M2e (MSLLTEVETPIRNEWGCRCNDSSD, IC) was amplified by PCR with primers p32F and p32R (Table S2). The DNA fragment coding for the seasonal influenza A (A/Western Australia/78/ 2005/H3N2, C17S and C19S) M2e mutation (MSLLTEVETPIRNEWGSRSNDSSD, ID) [1] was amplified by PCR with primers p32S24F and p32S24R, and M2e mutation (MSLLTEVEPIRNEWGSRSNDSSDPSR, IE) [1] was amplified by PCR with primers p32S27F and p32S27R (Table S2). The DNA fragment coding for the highly pathogenic avian influenza viruses (HPAIV) (A/Thailand/676/2005/H5N1) M2e (MSLLTEVETPTRNEWECRCSDSSD, IF) was amplified by PCR with primers p51F and p51R (Table S2). The DNA fragment coding for the HPAIV (A/Indonesia/CDC836T/2006/H5N1) M2e (MSLLTEVETPTRNEWECKCIDSSD, IG) was amplified by PCR with primers p51IF and p51IR (Table S2). The PCR-amplified fragments were purified and inserted into plasmid pBS-d35CP at the NheI and NotI sites.

The DNA fragment coding for the influenza A (A/South Carolina/1/18/H1N1) HA2 epitope A (IDGWYGYHHQN, IX) [2] was amplified by PCR with primers pBHA21F and pBHA21R, and HA2 epitope B (QKSTQNAIDGITNKVNSVI, IJ) [2] was amplified by PCR with primers pBHA22F and pBHA22R (Table S2). The PCR-amplified fragments were purified and inserted into plasmid pBS-d35CP at the NheI and NotI sites.

The DNA fragment coding for the influenza A (H5N1) HA epitope A (HEASSGVSSACPYQGKSSFFRNVVWLIKK, IM) was amplified by PCR with primers pHA29F and pHA29R, HA epitope B (GVSSACPYQGKSSFFRNVVWLIKK, IN) was amplified by PCR with primers pHA24F and pHA24R, HA epitope C (SSACPYQGKSSFFRN VVWLIKK, IO) was amplified by PCR with primers pHA22F and pHA24R, HA epitope D (A CPYQGKSSFFRNVVWLIKK, IP) was amplified by PCR with primers pHA20F and pHA24R, HA epitope E (PYQGKSSFF RNVVWLIKK, IQ) was amplified by PCR with primers pHA18F and pHA24R, and HA epitope F (KSSFFRNVVWLIKK, IR) was amplified by PCR with primers pHA14F and pHA24R, HA epitope G (SSACPYQGKSSFFRNVVWLI, IS) was amplified by PCR with primers pHA22F and pHA22R, HA epitope H (ACPYQGKSSFFRNV VWLI, IT) was amplified by PCR with primers pHA20F and pHA22R (Table S2). The PCR-amplified fragments were purified and inserted into plasmid pBS-d35CP at the NheI and NotI sites.

The DNA fragment coding for the JEV (CH2195LA) EDIII epitope A (DKLALKGTTYGM CTEKFSFAKNPADTGHGT, JA) was amplified by PCR with primers pD3N and pJ30R, EDIII epitope B (GDSYIVVGMGDKQINHHWHKAGST, JB) was amplified by PCR with primers pJ88N and pD3R (Table S2) using plasmid pET32a/LD3 [3] as template. The DNA fragment coding for the JEV (CH2195LA) EDIII epitope (GDSYIVVGRGD KQINH HWHKAGST, JC) was amplified by PCR with primers pJ88N and pD3R (Table S2) using plasmid pB2A [4] as template. The PCR-amplified fragments were purified and inserted into plasmid pBS-d35CP at the NheI and NotI sites.

The DNA fragment coding for the tissue plasminogen activator signal sequence (MKRGL CCVLLLCGAVFVS, LA) [5] was amplified by PCR with primers pTPAF and pTPAR (Table S2). The PCR-amplified fragments were purified and inserted into plasmid pBS-d35CP at the AgeI and NheI sites.

The DNA fragment coding for the FMDV type O (O/TAW/97) epitope (SSKYGDTSTN, FA) was amplified by PCR with primers pBVP197mF and pr21N, FMDV type O (O/TAW/99) epitope (NCKYGESPVT, FB) was amplified by PCR with primers pBVP199mF and pr21N, FMDV type A (A22 Iraq) epitope (TSKYSAGGTG, FC) was amplified by PCR with primers pBVP1A22mF and pr21N, FMDV type Asia 1 (As2/Tajikistan/1/2004) epitope (KTTYGETTRR,

FD) was amplified by PCR with primers pBVP1AS1mF and pr21N (Table S2) using plasmid pBVP1 [6] as template. The PCR-amplified fragments were purified and inserted into plasmid pBS-d35CP at the NheI and NotI sites.

The DNA fragment coding for the FMDV (O/TAW/99) VP1 part sequence (TVYNGNCKYGESPVTNVRGDLQVLAQKAARTLPTSFN, OF) was amplified by PCR with primers pVP199F and pVP199R (Table S2). The PCR-amplified fragments were purified and inserted into plasmid pBS-d35CP at the AgeI and NotI sites to give plasmid pBVP1-9.

The DNA fragment coding for the twice FMDV (O/TAW/97) VP1 part sequence (TVYNGSSKYGDTSTNNVRGDLQVLAQKAERTLPTSFNAAATVYNGSSKYGDTSTNNVRGDLQVLAQKAERTLPTSFN, OEE, spacer, underlined) was amplified by PCR with primers pVP1AF and pVP1R, the FMDV (O/TAW/97) and (O/TAW/99) VP1 part sequence (TVYNGNCKYGESPVTNVRGDLQVLAQKAARTLPTSFNAAATVYNGSSKYGDTSTNNVRGDLQVLAQKAERTLPTSFN, OFE, spacer, underlined) was amplified by PCR with primers pVP1AF and pVP1R, FMDV (O/TAW/97) and (O/TAW/99) VP1 part sequence (TVYNGNCKYGESPVTNVRGDLQVLAQKAARTLPTSFNAAASGGGGTVYNGSSKYGDTSTNNVRGDLQVLAQKAERTLPTSFN, OFE1, spacer, underlined) was amplified by PCR with primers pVP1AGF and pVP1R, and FMDV (O/TAW/97) and (O/TAW/99) VP1 part sequence (TVYNGNCKYGESPVTNVRGDLQVLAQKAARTLPTSFNAAATVYNGSSKYGDTSTNNVRGDLQVLAQKAERTLPTSFNAAATVYNGSSKYGDTSTNNVRGDLQVLAQKAERTLPTSFN, OFEE, spacer, underlined) was amplified by PCR with primers pVP1AF and pVP1R (Table S2) using plasmid pBVP1 [6] as template. The PCR-amplified fragments were purified and inserted into plasmid pBVP1 [6], pBVP1-9, pBVP1-9A7 at the NotI sites to give plasmid pBVP1-7A7, pBVP1-9A7, pBVP1-9AG7, and pBVP1-9A7A7, respectively.

The DNA fragment coding for the twice FMDV (O/TAW/99) VP1 part sequence (TVYNGNCKYGESPVTNVRGDLQVLAQKAARTLPTSFNAAATVYNGNCKYGESPVTNVRGDLQVLAQKAARTLPTSFN, OFF, spacer, underlined) was amplified by PCR with primers pVP1AF and pVP1R, the FMDV (O/TAW/97) and (O/TAW/99) VP1 part sequence (TVYNGSSKYGDTSTNNVRGDLQVLAQKAERTLPTSFNAAATVYNGNCKYGESPVTNVRGDLQVLAQKAARTLPTSFN, OEF, spacer, underlined) was amplified by PCR with primers pVP1AF and pVP1R, and FMDV (O/TAW/97) and (O/TAW/99) VP1 part sequence (TVYNGSSKYGDTSTNNVRGDLQVLAQKAERTLPTSFNAAASGGGGTVYNGNCKYGESPVTNVRGDLQVLAQKAARTLPTSFN, OEF1, spacer, underlined) was amplified by PCR with primers pVP1AGF and pVP1R (Table S2) using plasmid pBVP1-9 as template. The PCR-amplified fragments were purified and inserted into plasmid pBVP1-9 or pBVP1 [6] at the NotI sites to give plasmid pBVP1-9A9, pBVP1-7A9, and pBVP1-7AG9, respectively.

Table S2 The primers used in this study

Primer Name	Nucleotide sequence (5'-3')
p11F	$^{5'}$ATGGACTAGTACCATGAGTCTTCTAACCGAGGTCGAAACGCCTATCAGAAA$^{3'}$
p11R	$^{5'}$ATTGCGGCCGCATCACTTGAACCGTTGCATCTGCACCCCCATTCGTTTCTGATA$^{3'}$
p1109F	$^{5'}$ATGGACTAGTACCATGAGTCTTCTAACCGAGGTCGAAACGCCTACCAGAAG$^{3'}$
p1109R	$^{5'}$ATTGCGGCCGCATCACTTGAATCGCTGCATCTGCACTCCCATTCGCTTCTGGTA$^{3'}$
p32F	$^{5'}$CATGGACTAGTACCATGAGTCTTCTAACCGAGGTCGAAACGCCTATCAGAAA$^{3'}$
p32R	$^{5'}$ATTGCGGCCGCATCACTTGAATCGTTGCATCTGCACCCCCATTCGTTTCTGATA$^{3'}$
p32S24F	$^{5'}$ATGGACTAGTACCATGAGTCTTCTAACCGAGGTCGAAACGCCTATCAGAAA$^{3'}$
p32S24R	$^{5'}$ATTGCGGCCGCGTCTGAGCTATCGTTAGAGCGAATCCCCCATTCGTTTCTGATA$^{3'}$
p32S27R	$^{5'}$ATTGCGGCCGCTCTAGACGGGTCTGAGCTATCGTTAGAGCGAATCCCCCATTCGTTTCTGATA$^{3'}$
p51F	$^{5'}$ATGGACTAGTACCATGAGTCTTCTAACCGAGGTCGAAACGCCTACCAGAAA$^{3'}$
p51R	$^{5'}$ATTGCGGCCGCATCACTTGAATCGCTGCATCTGCATTCCCATTCGTTTCTGGTA$^{3'}$
p51IF	$^{5'}$ATGGACTAGTACCATGAGTCTTCTAACCGAGGTCGAAACGCCTACCAGAAA$^{3'}$
p51IR	$^{5'}$ATTGCGGCCGCATCACTTGAATCGATGCATTTGCACTCCCATTCGTTTCTGGTA$^{3'}$
pHA8.51F	$^{5'}$GGACTAGTACCATGTGCAACACCAAGTGTCAAACTCCAGCGGCCGCACAGCCCTGG $^{3'}$
pr21N	$^{5'}$GCGGGAGCTCTTTTTTTTTTTTTTT $^{3'}$
pBHA21F	$^{5'}$ GGACTAGTACCATGATAGATGGATGGTATGGTTAT $^{3'}$
pBHA21R	$^{5'}$ ATTGCGGCCGCATTCTGATGATGATAACC $^{3'}$
pBHA22F	$^{5'}$ GGACTAGTACCATGCAAAAAAGCACACAAAATGCCATTGACGGGATT $^{3'}$
pBHA22R	$^{5'}$ ATTGCGGCCGCGATAACAGAATTCACCTTGTTTGTAATCCCGTC $^{3'}$
pBIAHA29F	$^{5'}$GGACTAGTACCATGCATGAAGCCTCATCAGGGGTGAGCTCAGCATGTCCATACAATGGGAA$^{3'}$
pBIAHA29R	$^{5'}$ATTGCGGCCGCCTTTTTGATAAGCCATACCACATTTCTGAAAAAGGAGGACTTCCCAT$^{3'}$
pBIAHA24F	$^{5'}$GGACTAGTACCATGGGCGTGTCATCAGCCTGCCCATACCAAGGAAAGTCATCATTC$^{3'}$
pBIAHA24R	$^{5'}$ATTGCGGCCGCCTTTTTGATGAGCCATACCACGTTTCGGAAGAATGATGA$^{3'}$
pBIAHA22F	$^{5'}$GGACTAGTACCATGTCATCAGCCTGCCCATACCAAGGAAAGTCATCATTC$^{3'}$
pBIAHA20F	$^{5'}$GGACTAGTACCATGGCCTGCCCATACCAAGGAAAGTCATCATTC$^{3'}$
pBIAHA18F	$^{5'}$GGACTAGTACCATGCCATACCAAGGAAAGTCATCATTC$^{3'}$
pBIAHA14F	$^{5'}$GGACTAGTACCATGAAGTCATCATTC$^{3'}$
pBIAHA22R	$^{5'}$ATTGCGGCCGTGATGAGCCATACCACGTTTCGGAAGAATGATGA$^{3'}$
pBVP197mF	5' AGCTAGCATGAGCAGTAAGTACGGTGACACCAGCACTAACGCGGCCGCGCTGTT 3'
pBVP199mF	5' AGCTAGCATGAACTGCAAGTATGGCGAGAGCCCCGTGACCGCGGCCGCGCTGTT 3'
pBVP1AS1mF	5' AGCTAGCATGAAGACGACGTACGGGGAAACAACCCGACGGGCGGCCGCGCTGTT 3'
pBVP1A22mF	5' AGCTAGCATGACGAGCAAGTACTCCGCAGGTGGTACGGGCGCGGCCGCGCTGTT 3'
prJE-D3N	$^{5'}$GGACTAGTACCATGGACAAACTGGCCCTGAAAGGC $^{3'}$
pJ30R	$^{5'}$ATTGCGGCCGCCGCGAACGAG$^{3'}$
pJ88N	$^{5'}$GGACTAGTGGAGACTCCTAC$^{3'}$
prJE-D3R	$^{5'}$ ATTGCGGCCGCCGTGCTTCCTGCTTTGTG$^{3'}$
pTPAF	$^{5'}$TGACCGGTATGAAGAGAGGGCTCTGCTGTGTGCTGCTGCTGTGTGGA$^{3'}$
pTPAR	$^{5'}$CGCGCTAGCCGAAACGAAGACTGCTCCACA$^{3'}$
pVP199F	$^{5'}$GATGACCGGTGCTAGTACCATGGACACTGTTTACAACGGGAACTGCAAGTATGGCGAGAGCCCCGTGACCAACGTGAGA$^{3'}$
pVP199R	$^{5'}$CTGTGCGGCCGCGTTGAAGGAGGTAGGCAGCGTTCTTGCCGCCTTCTGGGCCAATACTTGCAGGTCACCTCTCACGTTG$^{3'}$
pVP1AF	$^{5'}$CAACGCGGCCGCAACCGTCTACAACGGG $^{3'}$
pVP1R	$^{5'}$ TGTGCGGCCGCACAGTTGAAGGAGGTAG$^{3'}$
pVP1AGF	$^{5'}$CAACGCGGCCGCATCTGGTGGCGGAGGCACCGTCTACAACGGG $^{3'}$

Methods S2. PVX chimeric particles produce antibodies for the distinction between FMDV O strain topotypes.

1. Comparison of amino acid sequences between the BaMV and PVX CP

PVX has a 6.4 kb single-stranded positive-sense RNA genome which is coated by approximately 1300 units of the same viral CP-generating filamentous particles. PVX-based vector (pPVX201) were steady transient expression of foreign genes in plants [7,8], and an N-terminal fusion with the CP was generated on the chimeric virus particle surface as described previously [9]. In addition, the complete genomic sequences of only 17 PVX isolates have been determined [10] and classified the PVX isolates into two groups Eurasia and America that correspond with their geographical origins [11]. We compared the CP sequence of one PVX isolate from Taiwan in 2000 (GenBank ID: AF272736.2) with PVX201 (GenBank ID: AAA03493.1), and there were only two amino acids (positions 10 and 11) different among the CP (Figure S1).

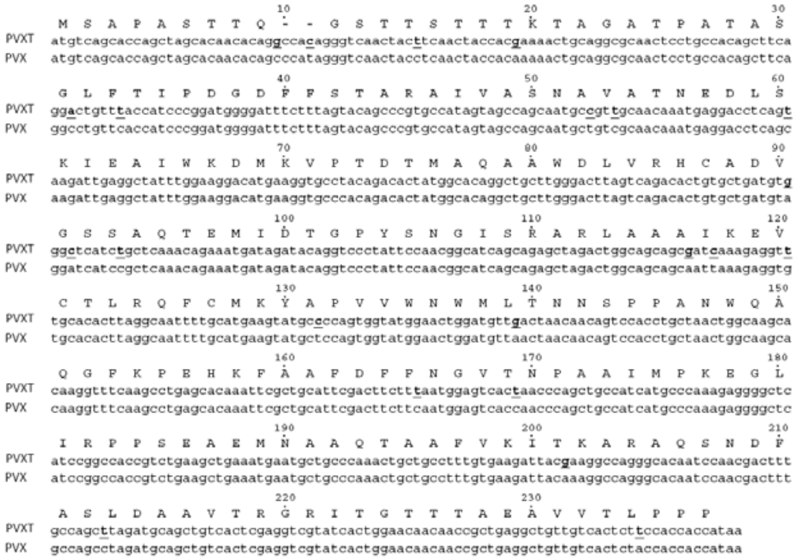

Figure S1. Comparison of the nucleotide and amino acid sequences of the PVX coat protein (CP) (GenBank ID: AAA03493.1) and PVX Taiwan strain (PVX-T) CP (GenBank ID: AF272736.2). The deduced conserved amino acid sequence is presented upper the coding of the exons and nucleotide difference sites are bold in below line.

The alignment of the deduced amino acid sequences and secondary structures for the entire CP of BS-d35CP and PVX is shown in Figure S2. Thence, the ALIGN calculates a global alignment of two sequences was used to alignment the sequences [12], and PSIPRED Protein Structure Prediction Server was used to predict protein secondary structures [13]. The alignment parameters that scoring matrix were BLOSUM50 and gap penalties were –12/–2. The BaMV and PVX CP amino acid sequences was through the ALIGN calculates a global alignment assay have been determined to 23.7% identity (Figure S2). Moreover, the BaMV and PVX CP secondary structure prediction results were similar (Figure S2).

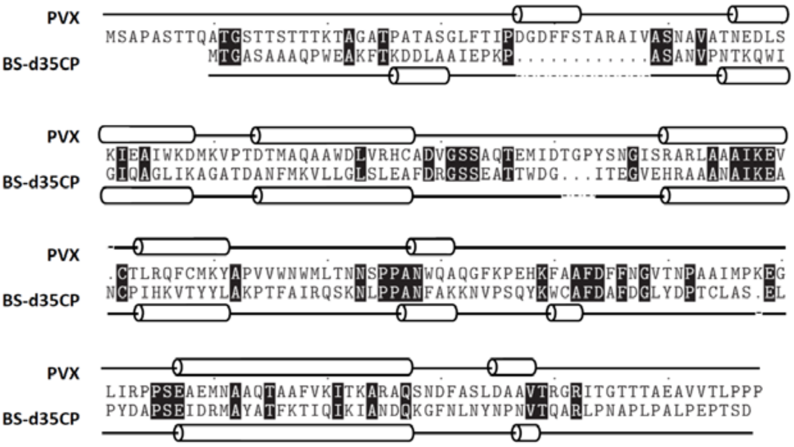

Figure S2. Comparison of the amino acid sequences and secondary structure of the PVX Taiwan strain (PVX-T) coat protein (CP) (GenBank ID: AF272736.2) and BS-d35CP. Identical amino acids residues are shown as white letters on a black background. Secondary structure assignments are as follows: black line, coil; bar, α-helix.

2. Construction and characterization of PVX-T CP mutants

The full-length infectious cDNA of PVX-T (GenBank ID: AF272736) with an upstream *Cauliflower mosaic virus* 35S promoter sequence was cloned in the plasmid pUC119. In addition, the PVX CP residues 2–22 at the N-terminus were lost occasionally. The N-terminus 22 amino acids deletion of PVX CP virus after repeated infection cycles, the sequence of the viral genome did not change and did not substantially affect cell-to-cell movement [14]. Therefore, the PVX-T CP N-terminus 22-amino acid sequence deletion mutants were constructed by restriction digestion pPVX-T with NheI (correspondint to PVX-T 5662 nt) and XhoI (corresponding to PVX-T 6302 nt), and replaced with the PCR-amplified CP fragments, digested with NheI and XhoI, corresponding to delete 22 amino acids from CP N-terminus. The primers used were $^{5'}$gcgctagccatggcggccgcaatgtcagcaccagcaagc acaacacaggcc$^{3'}$ and $^{5'}$gcgggagctctttttttttttttt$^{3'}$ (with PVX-T CP coding sequences italicized, and restriction sites for NheI and XhoI, respectively, underlined) by using pPVX-T as template. The mutant was denoted as pPT-d22CP (Figure S3a). The chimeric VP1-97s-CP were amplified by used $^{5'}$agcta gcatg**agcagtaagtacggtgacaccagcactaac**gcggccgcaggcgc$^{3'}$ and $^{5'}$gcgggagctctttttttttttttt$^{3'}$ (with PVX-T CP coding sequences italicized, FMDV VP1 part coding sequences in boldface, and restriction sites for NheI and XhoI, respectively, underlined) by using pPT-d22CP as template. The chimeric VP1-99s-CP were amplified by used $^{5'}$agctagcatg**aactgcaagtatggcgagagccccgtgac c**gcggccgcaggcgc$^{3'}$ and $^{5'}$gcgggagctct ttttttttttttt$^{3'}$ (with PVX-T CP coding sequences italicized, FMDV VP1 part coding sequences in boldface, and restriction sites for NheI and XhoI, respectively, underlined) by using pPT-d22CP as template. The above-mentioned chimeric VP1-97s-CP and VP1-99s-CP coding sequences were inserted into plasmid pPVX-T at the NheI and XhoI sites to give plasmid pPVP1-97s and pPVP1-99s, respectively (Figure S3b and S3c).

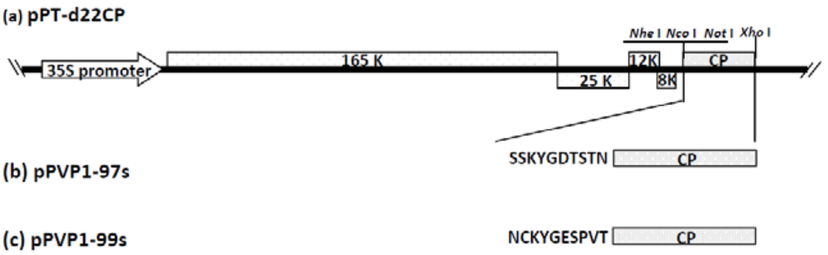

Figure S3. Schematic representation of the recombinant pPVP1-97s and pPVP1-99s based on PVX genome. The pPT-d22CP is a mutant infectious construct of PVX with 22 N-terminal amino acids

truncation in its coat protein (CP), under the control of Cauliflower mosaic virus 35S promoter. The pPVP1-97s and pPVP1-99s are recombinant plasmids derived from pPT-d22CP by inserting the DNA coding for the FMDV VP1 region, to replace that coding for the N-terminal coat protein (CP). The pPVP1-97s derived from amino acid position 133–142 of VP1 of FMDV O/TAW/97 strain, and the pPVP1-99s derived from amino acid position 133–142 of VP1 of FMDV O/TAW/99 strain. The positions of open reading frames and the proteins encoded were indicated by the boxes. The restriction enzyme recognition sites for cloning are indicated on top.

3. Production of specific FMDV VP1 peptides using chimeric PVX-T in Plants

To verify the ability of pPVP1-97s and pPVP1-99s to produce infectious virus particles, the recombinant vectors were used to inoculate *N. benthamiana* and *N. tabacum 'vanHicks'* plants grown in greenhouse with normal daylight. Plants inoculated leaves were harvested 7 dpi, and systemic leaves were harvested 10 dpi. Typically, six plants of *N. benthamiana* with two leaves per plant were inoculated for systemic symptom observation at 14 dpi. Four plants of *N. tabacum* with two leaves per plant were inoculated with DNA for systemic symptom observation at 10 dpi. The *N. benthamiana* or *N. tabacum* systemic leaves of the plants displayed the same symptoms induced by pPVP1-97s and pPVP1-99s (Figure S4a). The *N. benthamiana* leaves from plants showing inoculation were collected, and the viruses were verified by western immunoblot analysis and Coomassie Blue staining (CBS) (Figure S4b). Total proteins extracted from pPVP1-97s and pPVP1-99s inoculated leaves via 12% SDS-PAGE separation and CBS, the results showed at the predicted molecular mass of 24.1 and 24.2 kDa obvious increasing to the pPVP1-97s and pPVP1-99s CP (Figure S4b). Then the total proteins extract of pPVP1-97s and pPVP1-99s inoculated leaves reacted with rabbit antiserum against PVX CP and that the reacting chimaeric proteins migrated at lower molecular weights than PT-d22CP, at 24.1 and 24.2 kDa consistent with MW estimates of the fused peptides. When the pPVP1-97s and pPVP1-99s were inoculated to *N. tabacum*, inoculated leaf analysis results similar to those observed in *N. benthamiana* were obtained (Figure S4b). To verify the presence of chimeric virus in systemically infected leaves of *N. benthamiana* or *N. tabacum*, total protein was extracted from plants infected with individual pPVP1-97s and pPVP1-99s (Figure S4b). Western blot results from systemically infected *N. benthamiana* or *N. tabacum* leaves agreed with those of inoculated *N. benthamiana* or *N. tabacum* leaves in that each chimeric virus migrated at lower molecular weights than PT-d22CP, at 24.1and 24.2 kDa consistent with MW estimates of the fused peptides. Taken together, these results indicated that the FMDV VP1 partial sequence fusions to the N-terminus of the CP no affected systemic movement of PT-d22CP.

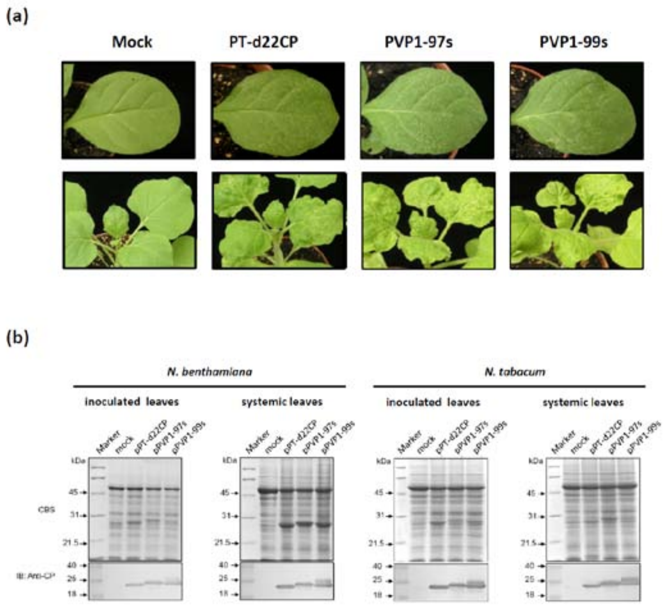

Figure S4. Symptoms and molecular analysis of plants inoculated with the potato virus X viral expression vectors. (a) Symptoms of the systemic leaves of *N. tabacum* plants (top panel) and *N. benthamiana* plants (bottom panel) inoculated with ddH_2O, pPT-d22CP, pPVP1-97s and pPVP1-99s. (b) The accumulation PVX viral expression vectors in plants. SDS-PAGE separation and immunoblotting of proteins extracted from *N. benthamiana* inoculated leaves at 7 dpi and systemic leaves at 7 dpi. The accumulation PVX viral expression vectors in. SDS-PAGE separation and immunoblot of proteins extracted from *N. tabacum* inoculated leaves at 7 dpi and systemic leaves at 10 dpi. Proteins extracted from leaves infected with vector pPT-d22CP, pPVP1-97s, and pPVP1-99s were electrophoresed with 12% SDS-PAGE. The upper panels of each figure were gels stained with Coomassie Blue stain (CBS), the lower panel were western blots (IB) immuno-stained with antisera against PVX CP. The relative molecular weights (in kDa) are given on the left of each panel.

4. Chimeric PVX-T particles produce antibodies for the distinction between FMDV O/TAW/97 strain and O/TAW/99 strain

For chimeric virus purification, *N. benthamiana* plants that had been inoculated with pPVP1-97s and pPVP1-99s were harvested systemic leaves at 10 dpi, respectively. The virions were subsequently purified from the leaves and the yield was determined by ultraviolet absorption as described previously [15]. Preparation of FMDV O/TAW/97 or O/TAW/99 strain VP1 antiserum purified chimeric PVP1-97s virions or PVP1-99s virions were diluted to a concentration of 1 mg/ml and mixed with an equal volume of Freund's incomplete adjuvant. Intraperitoneal (i.p.) injection of ICR mice were performed one dose by using 0.1 ml of immunogen. Mice serum samples were bled via the periorbital route, collected 3, 4, and 5 weeks after the first immunization and used for immunoblotting analyses as described below. Then the BVP1 and BVP1-9 virions reacted with rabbit antiserum against BaMV CP, rabbit antiserum against FMDV VP1 [16], mice antiserum against FMDV (O/TAW/97) VP1, and mice antiserum against FMDV (O/TAW/99) VP1. The results showed that immunized mice elicited VP1-specific antibody for the distinction between FMDV O/TAW/97 and O/TAW/99 strain (Figure S5).

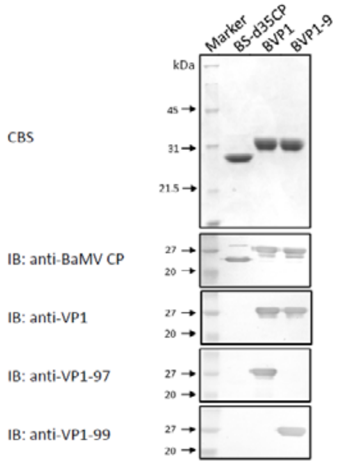

Figure S5. PVX chimeric particles produce antibodies for the distinction between FMDV O strain topotypes. The BVP1 and BVP1-9 virions were separated in a 12% SDS-PAGE, stained with CB. The proteins were transferred to PVDF membranes and reacted with antisera against BaMV CP (anti-CP), FMDV-O VP1 (anti-VP1), FMDV VP1 O/TAW/97 (anti-VP1-97), and FMDV VP1 O/TAW/99 (anti-VP1-99), respectively. Lane 1, protein molecular markers; lane 2; BS-d35CP virion; lane 3, BVP1 virion; lane 4, BVP1-99 virion. The relative molecular weights (in kDa) are given on the left of each panel.

References

1. Mozdzanowska, K.; Feng, J.; Eid, M.; Kragol, G.; Cudic, M.; Otvos, L., Jr.; Gerhard, W. Induction of influenza type A virus-specific resistance by immunization of mice with a synthetic multiple antigenic peptide vaccine that contains ectodomains of matrix protein 2. *Vaccine* **2003**, *21*, 2616-2626.
2. Sui, J.; Hwang, W.C.; Perez, S.; Wei, G.; Aird, D.; Chen, L.M.; Santelli, E.; Stec, B.; Cadwell, G.; Ali, M., et al. Structural and functional bases for broad-spectrum neutralization of avian and human influenza A viruses. *Nat Struct Mol Biol* **2009**, *16*, 265-273.
3. Wu, S.C.; Yu, C.H.; Lin, C.W.; Chu, I.M. The domain III fragment of Japanese encephalitis virus envelope protein: mouse immunogenicity and liposome adjuvanticity. *Vaccine* **2003**, *21*, 2516-2522.
4. Chen, T.H.; Hu, C.C.; Liao, J.T.; Lee, Y.L.; Huang, Y.W.; Lin, N.S.; Lin, Y.L.; Hsu, Y.H. Production of Japanese Encephalitis Virus Antigens in Plants Using Bamboo Mosaic Virus-Based Vector. *Front Microbiol* **2017**, *8*, 788.
5. Li, Z.; Howard, A.; Kelley, C.; Delogu, G.; Collins, F.; Morris, S. Immunogenicity of DNA vaccines expressing tuberculosis proteins fused to tissue plasminogen activator signal sequences. *Infect Immun* **1999**, *67*, 4780-4786.
6. Yang, C.D.; Liao, J.T.; Lai, C.Y.; Jong, M.H.; Liang, C.M.; Lin, Y.L.; Lin, N.S.; Hsu, Y.H.; Liang, S.M. Induction of protective immunity in swine by recombinant bamboo mosaic virus expressing foot-and-mouth disease virus epitopes. *BMC Biotechnol* **2007**, *7*, 62.
7. Chapman, S.; Kavanagh, T.; Baulcombe, D. Potato virus X as a vector for gene expression in plants. *Plant J* **1992**, *2*, 549-557.
8. Baulcombe, D.C.; Chapman, S.; Santa Cruz, S. Jellyfish green fluorescent protein as a reporter for virus infections. *Plant J* **1995**, *7*, 1045-1053.
9. Marusic, C.; Rizza, P.; Lattanzi, L.; Mancini, C.; Spada, M.; Belardelli, F.; Benvenuto, E.; Capone, I. Chimeric plant virus particles as immunogens for inducing murine and human immune responses against human immunodeficiency virus type 1. *J Virol* **2001**, *75*, 8434-8439.
10. Yu, X.Q.; Jia, J.L.; Zhang, C.L.; Li, X.D.; Wang, Y.J. Phylogenetic analyses of an isolate obtained from potato in 1985 revealed potato virus X was introduced to China via multiple events. *Virus Genes* **2010**, *40*, 447-451.
11. Yu, X.Q.; Wang, H.Y.; Lan, Y.F.; Zhu, X.P.; Li, X.D.; Fan, Z.F.; Li, H.F.; Wang, Y.Y. Complete Genome Sequence of a Chinese Isolate of Potato virus X and Analysis of Genetic Diversity. *J Phytopathol* **2008**, *156*, 346-351.
12. Myers, E.W.; Miller, W. Optimal alignments in linear space. *Comput Appl Biosci* **1988**, *4*, 11-17.
13. McGuffin, L.J.; Bryson, K.; Jones, D.T. The PSIPRED protein structure prediction server. *Bioinformatics* **2000**, *16*, 404-405.
14. Lico, C.; Capuano, F.; Renzone, G.; Donini, M.; Marusic, C.; Scaloni, A.; Benvenuto, E.; Baschieri, S. Peptide display on Potato virus X: molecular features of the coat protein-fused peptide affecting cell-to-cell and phloem movement of chimeric virus particles. *J Gen Virol* **2006**, *87*, 3103-3112.
15. Lin, N.S.; Chen, C.C. Association of bamboo mosaic virus (BaMV) and BaMV-specific electron-dense crystalline bodies with chloroplasts. *Phytopathology* **1991**, *81*, 1551-1555.
16. Wang, J.H.; Liang, C.M.; Peng, J.M.; Shieh, J.J.; Jong, M.H.; Lin, Y.L.; Sieber, M.; Liang, S.M. Induction of immunity in swine by purified recombinant VP1 of foot-and-mouth disease virus. *Vaccine* **2003**, *21*, 3721-3729.
